# Supplementary material for: Gastrodin alleviates neuronal damage in epileptic cell models by targeting P2RY12 to inhibit microglial hyperactivation
Source: PLoS One. 2026 Apr 17;21(4):e0346877. doi: 10.1371/journal.pone.0346877 (PMC13089871; doi:10.1371/journal.pone.0346877)
Supplement: S1 File — S1 Fig. Validation of the knockdown model for P2RY12. a. qPCR assay was used to detect the knockdown of P2RY12 mRNA in HMC3 microglial cells, with n = 3.b-c. Western blot assay was employed to verify the downregulation of P2RY12 protein expression in HMC3 microglial cells, with n = 3. Supplementary Table 1. Primer sequence table. The sequences of qPCR primers used in this study. (DOCX) [file pone.0346877.s001.docx]

**Supplementary Materials for**

Gastrodin Alleviates Neuronal Damage in Epileptic Cell Models by Targeting P2RY12 to Inhibit Microglial Hyperactivation.

Aiyuan Cai^1^, Zilong Li^1^, Ran Liu^1^, Hailong Huang^1^, Ping Liu^1^, Ruizhong Zhang^1^, Jing Xiao^1^, Yuanhong Lin^2^, Qingpeng Hu^1*^, Haixia Wu^1*^

*^1^Department of Pediatrics, Longhua District People's Hospital, Shenzhen, Guangdong, 518109, China*

*^2^Second Clinical Medical College, Guangzhou University of Chinese Medicine, Guangzhou 510410*

*Corresponding authors:

Qingpeng Hu: huqingpeng163@126.com

Haixia Wu: [13923430437@163.com](mailto:13923430437@163.com)

1. Supplementary Figure 1. Validation of the knockdown model for P2RY12.


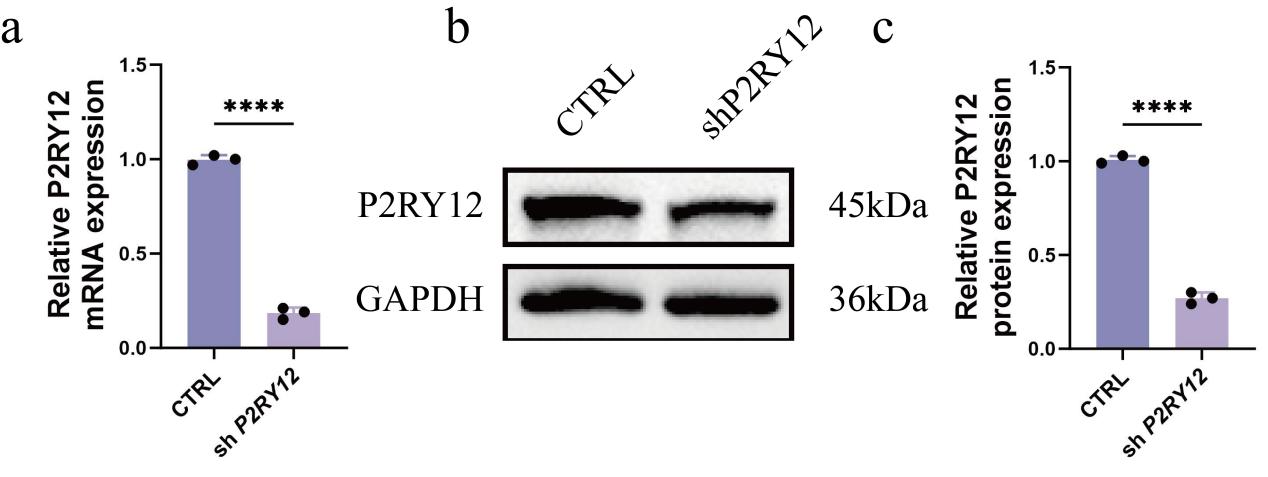


a. qPCR assay was used to detect the knockdown of *P2RY12* mRNA in HMC3 microglial cells, with n = 3.

b-c. Western blot assay was employed to verify the downregulation of P2RY12 protein expression in HMC3 microglial cells, with n = 3.

1. Supplementary Table1

| Species | Primer  name | Amplicon  size | F/R | Sequence 5’-3’ | Length | Location |
| --- | --- | --- | --- | --- | --- | --- |
| Homo sapiens | P2RY12 | 163 | Forward | CAGAAGACAGGAGCTGCAGAA | 21 | 6-26 |
|  |  |  | Reverse | CTGCAGAGTGGCATCTGGTAT | 21 | 168-148 |
| Homo sapiens | β-actin | 70 | Forward | CTGGAACGGTGAAGGTGACA | 20 | 1395-1378 |
|  |  |  | Reverse | TTCACGTTTGCGGTTTGGTT | 20 | 1051-1032 |
