# Supplementary material for: Gastrodin alleviates neuronal damage in epileptic cell models by targeting P2RY12 to inhibit microglial hyperactivation
Source: PLoS One. 2026 Apr 17;21(4):e0346877. doi: 10.1371/journal.pone.0346877 (PMC13089871; doi:10.1371/journal.pone.0346877)
Supplement: S1 Raw image — The figure captions in the document, in order from top to bottom, are: a-b Correspond to Figure 4a in the original text; c-d Correspond to Figure 4b in the original text. a-b Correspond to Figure 4c in the original text. a-c Correspond to Figure 7a in the original text; d Correspond to Supplementary Figure 1b in the supplementary material text. (PDF) [file pone.0346877.s002.pdf]

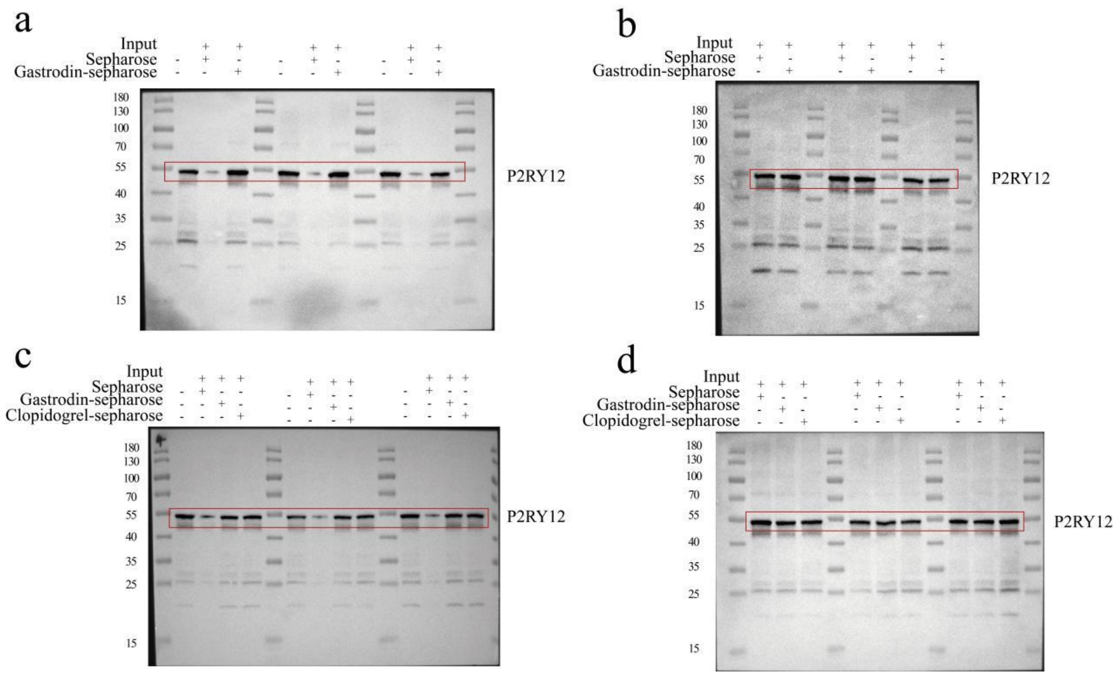

Presentation of Full Membrane Results from Western Blot Experiments

a-b Correspond to Figure 4a in the original text; c-d Correspond to Figure 4b in the original text.

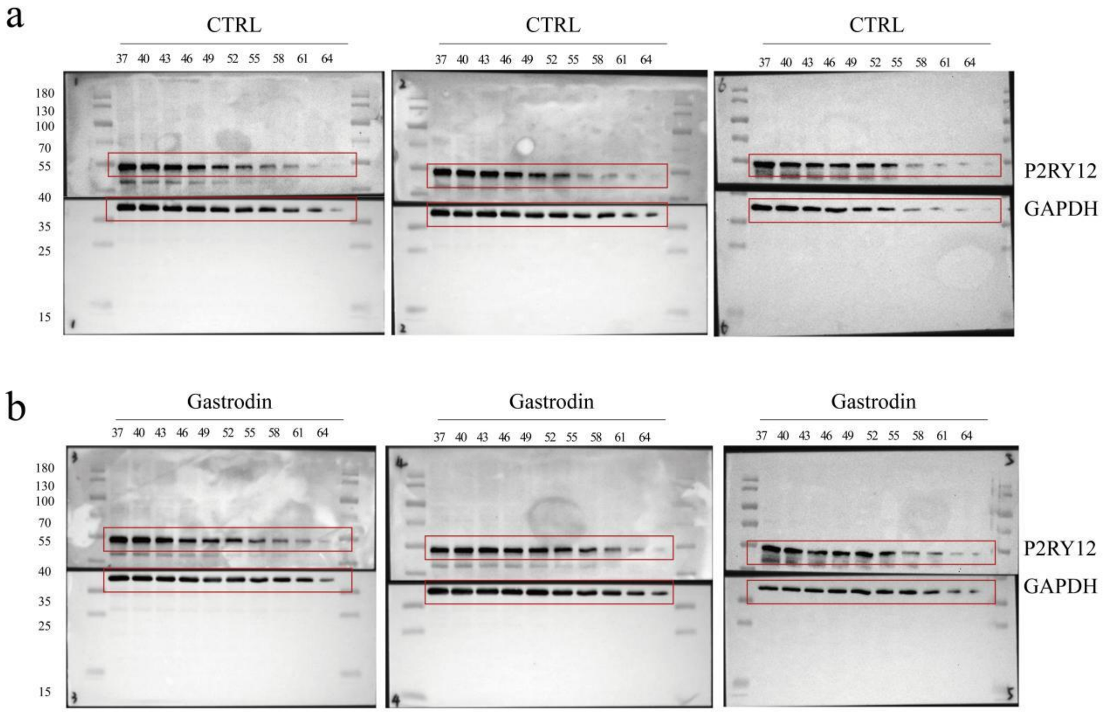

Presentation of Full Membrane Results from Western Blot Experiments

a-b Correspond to Figure 4c in the original text.

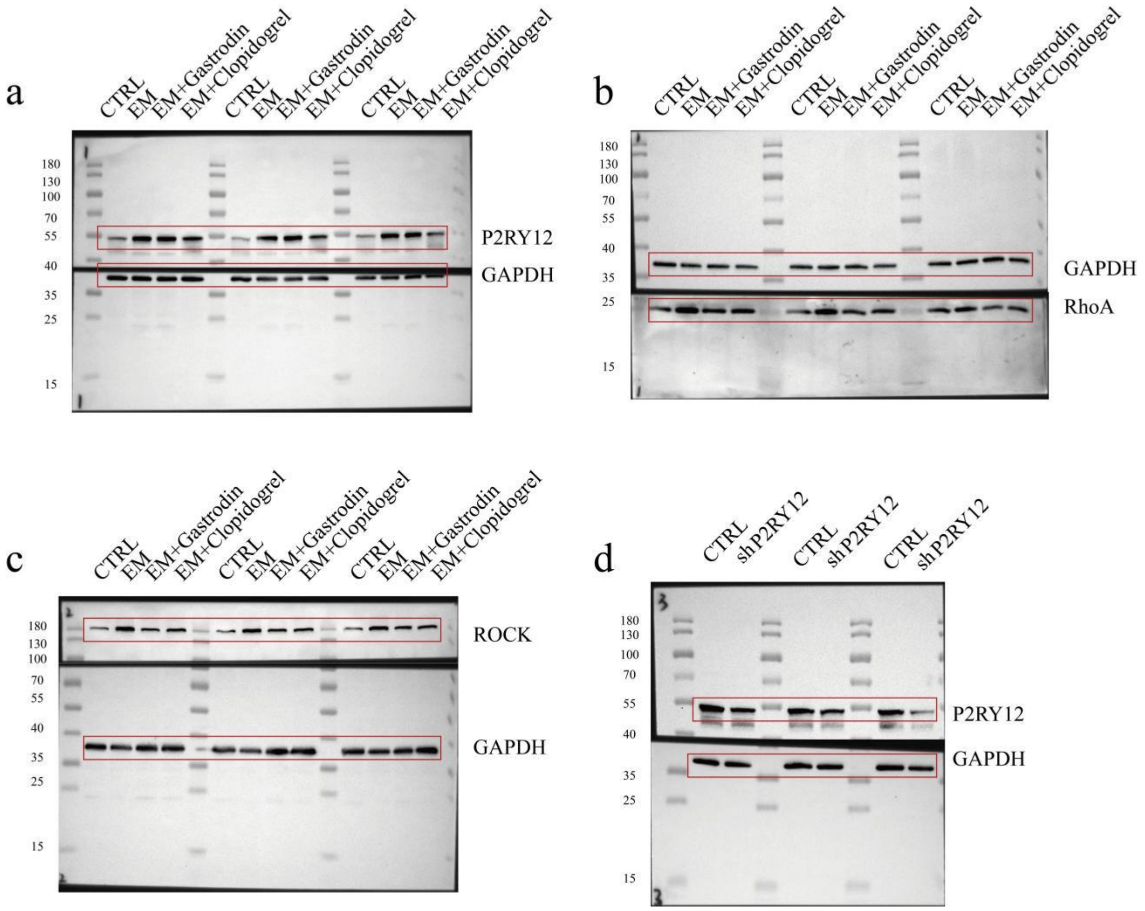

Presentation of Full Membrane Results from Western Blot Experiments

a-c Correspond to Figure 7a in the original text; d Correspond to Supplementary Figure 1b in the supplementary material text.
