# Supplementary material for: Gastrodin alleviates neuronal damage in epileptic cell models by targeting P2RY12 to inhibit microglial hyperactivation
Source: PLoS One. 2026 Apr 17;21(4):e0346877. doi: 10.1371/journal.pone.0346877 (PMC13089871; doi:10.1371/journal.pone.0346877)
Supplement: Attachment — Submitted filename: Review.docx [file pone.0346877.s003.docx]

**I. Terminology and Nomenclature**

1. The study focuses on gastrodin, not the whole herb *Gastrodia elata*. To avoid confusion, please replace all instances of “*Gastrodia elata*” in the title and text with “gastrodin”.
2. On line 712, “antiseizure medications (ASMs)” should be capitalized as “Antiseizure medications (ASMs)”.
3. Hyphens in compound terms (e.g., “drug-refractory epilepsy”) appear with spaces (e.g., “X - Y”). These should be closed up: “X-Y”.
4. On line 30, “Pull-down” should be lowercase (“pull-down”), as it is a standard assay name, not a proper noun.

**II. Experimental Details and Data Presentation**

5. The forward primer for β-actin is listed as “1395–1378”. Genomic coordinates should run 5′→3′; please correct to “1378–1395”.
6. The catalog number for the P2RY12 antibody is given only on its second mention. It should appear at first use—typically in the Western blot or immunofluorescence methods section.
7. The shRNA sequences used for P2RY12 knockdown are missing from the Methods. Please provide the target sequence(s) and details of the control shRNA.
8. Line 234 refers to “lung tissue supernatants”, but the study uses only HMC3/SH-SY5Y co-cultures. This appears to be an error; please remove or correct.
9. Line 605 states that EGFP expression decreased in the EM+Gastrodin+shP2RY12 group. If EGFP is a constitutive reporter (e.g., driven by *CX3CR1* or *Iba1*), its level should not vary with treatment. Clarify whether EGFP is part of the shRNA vector or a stable marker.

**III. Mechanistic Interpretation**

10. P2RY12 belongs to the P2Y receptor family (which includes P2Y6, P2Y13, etc.). The study does not address whether gastrodin affects other P2Y receptors.
11. Lines 39–40 describe P2RY12 “expression was interfered with”—a vague phrase. Specify the method: e.g., “after P2RY12 knockdown” or “following shRNA-mediated silencing”.

**IV. Figures and Formatting**

12. Figure fonts are inconsistent, and gene/protein italicization is incorrect. Remember: – Gene symbols (*P2RY12*) in italics; – Protein names (P2RY12) in roman type; Use a single font (e.g., Arial) and consistent size throughout all figures.

**V. References**

13. References [26] and [46] appear to cite the same paper. Keep one and ensure consistent author formatting.
14. The citation for Dai J et al. (2024) lacks volume, issue, and page numbers. Please complete the reference.
15. Reference [4] (Feigin et al., *Lancet Public Health* 2025) lists pages as “e203–e27”. The correct format is “e203–e227”. Also verify the author list against the original publication.

**VI. Ethical Statement**

16. The funding statement mentions “epileptic rats”, but no animal work is reported here—only cell cultures and database analysis. If the rat studies are part of separate projects, please add a note clarifying that no animals were used in this particular study.
