# Supplementary material for: Gastrodin alleviates neuronal damage in epileptic cell models by targeting P2RY12 to inhibit microglial hyperactivation
Source: PLoS One. 2026 Apr 17;21(4):e0346877. doi: 10.1371/journal.pone.0346877 (PMC13089871; doi:10.1371/journal.pone.0346877)
Supplement: Attachment — Submitted filename: 3.20Point by point response letter(1).docx [file pone.0346877.s005.docx]

Dear Xiaona Wang, Ph.D,

Thank you very much for considering our manuscript entitled "Gastrodin Alleviates Neuronal Damage in Epileptic Cell Models by Targeting P2RY12 to Inhibit Microglial Hyperactivation" (Manuscript number: PONE-D-25-64129) for publication in PLOS One. We are grateful to both the reviewers and the editor for their constructive comments and suggestions, which have greatly helped us improve the quality of the paper. We have carefully considered all the comments and have made the appropriate revisions to the manuscript. Below, we provide detailed responses to each of the points raised:

Response to Academic Editor

The funding information has been completely removed from the main text. We confirm that all funding‑related content has been deleted from the manuscript, and the funding declaration is only provided via the online submission system. The relevant statement has been added and updated in the cover letter accordingly.

**Figures and blots/gels:** All figures have been removed from the main text file and uploaded separately as TIFF files. All original uncropped, unadjusted blot/gel images are provided in the Supporting Information.

**Response to Reviewer #1**

Comment: The description of statistical methods lacks sufficient detail. Please specify the exact statistical test used for each comparison, and clarify whether assumptions of normality and homogeneity of variance were assessed.

Response:We sincerely appreciate the valuable comments and suggestions you have provided on our manuscript. We deeply apologize for the inadequate description of the statistical methods. We have thoroughly revised the section "2.12 Data Statistical Methods" to clearly state the following: the Shapiro-Wilk test is employed for normality testing, the Levene test is used for homogeneity of variance testing, an unpaired two-tailed t-test is applied for comparisons between two groups, one-way analysis of variance followed by Tukey's test is conducted for multiple-group comparisons, and for non-parametric data, the Kruskal-Wallis test combined with Dunn's test is utilized. The relevant modifications have been made in lines 316 to 324.

**Response to Reviewer #2**

Comment 1: The title is inaccurate. “Gastrodia elata” and “chronic epilepsy” are not sufficiently accurate.

Response:We are extremely grateful for your strict comments on the inaccuracy of the original title. In response to your suggestions, we have carefully revised the title to precisely define the research subject, experimental system and core mechanism. The revised title is as follows: Gastrodin Alleviates Neuronal Damage in Epileptic Cell Models by Targeting P2RY12 to Inhibit Microglial Hyperactivation.

Comment 2: Lines 348–349: Explain Figure 1d in detail; change colors of Figure 1d and Figure 2e for clarity.

Response:We sincerely thank you for your constructive suggestions on improving the presentation of the charts and graphs. We have supplemented a detailed description and interpretation of Figure 1d in lines 345 to 351 of the Results section. Additionally, the color schemes of Figure 1d (a Venn diagram) and Figure 2e have been adjusted to high-contrast light tones to enhance visual clarity and readability.

Comment 3: Figure 8 title fails to reflect the regulatory mechanism of Gastrodin; needs improvement.

Response:We sincerely appreciate your constructive feedback. To enhance clarity and accuracy, we have revised the title of Figure 8 to: "The mechanism by which gastrodin targets P2RY12 to inhibit the RhoA/ROCK pathway and microglial migration in an epilepsy model."

Comment 4: Paragraph lines 682–693 and the last sentence of the conclusion lack objectivity and rigor.

Response: We sincerely appreciate this constructive suggestion. We have carefully revised the paragraph spanning lines 682 to 693, as well as the last sentence of the conclusion section, to eliminate speculative and exaggerated expressions. All descriptions are now strictly based on the data and results obtained in this study, and the scientific objectivity and rationality of the conclusions have been significantly enhanced. Now, all conclusions are fully supported by our experimental evidence. The relevant modifications have been made in lines 677 to 693.

Comment 5: Errors in symbols (e.g., CO₂) and inconsistent capitalization of Gastrodin.

Response: We sincerely appreciate your meticulous corrections. We have carefully examined and revised the entire manuscript: the incorrect expression "CO2" has been uniformly corrected to "CO₂", and the capitalization of "Gastrodin" has been consistently maintained throughout the text to ensure formatting uniformity. All symbol and capitalization errors have been duly corrected.

We hope that the revisions and clarifications we have made will satisfactorily address the concerns raised by the reviewers. We would be more than willing to make further adjustments if necessary.

Thank you again for your consideration and for the opportunity to submit our work to PLOS ONE.

Sincerely,

Qingpeng Hu: huqingpeng163@126.com

Haixia Wu: 13923430437@163.com
